# The Brief Case: Suspicious Gram-Negative Coccobacilli—Francisella tularensis subsp. *novicida* Isolated from an Immunocompromised Patient

**DOI:** 10.1128/jcm.00787-22

**Published:** 2023-06-20

**Authors:** Kenneth Gavina, Brynne E. Whitacre, Thomas L. Meyer, Kayla Van Benten, Mark Glazier, Christopher L. Emery, John-Paul Lavik, Ryan F. Relich

**Affiliations:** a Department of Pathology and Laboratory Medicine, Indiana University School of Medicine, Indianapolis, Indiana, USA; b Indiana University Health, Indianapolis, Indiana, USA; c Indiana Department of Health Laboratories, Indianapolis, Indiana, USA; Pattern Bioscience

**Keywords:** Francisella tularensis, bioagent, biosafety, select agent, tularemia

## CASE PRESENTATION

A 63-year-old male with a history of diabetes, temporal arteritis, inflammatory polymyopathy, and previous diagnosis of blastomycosis presented with complaints of severe abdominal and back pain. Computed tomography scans of his abdomen and pelvis revealed moderate diffuse ascites, loculated fluid collections along the margin of the liver, and pancreatic pseudocyst formation. The patient was admitted for suspected acute pancreatitis, and notable laboratory test results were as follows: white blood cells 31.7 k/μL (normal range: 3.6 to 10.6 k/μL), neutrophils 29.3 k/μL (normal range: 1.7 to 7.5 k/μL), anion gap 14 mg/dL (normal range: 8 to 12 mg/dL), blood urea nitrogen 43 mg/dL (normal range: 5 to 20 mg/dL), C-reactive protein 1.2 mg/dL (normal range: 0.3 to 1.0 mg/dL), alkaline phosphatase 137 U/L (normal range: 25 to 125 U/L), and lipase 891 U/L (normal range: 24 to 151 U/L). Additional history included outdoor living, dog ownership, and tobacco use. Sexual history, alcohol consumption, and illicit drug-use were not reported. Two sets of blood cultures (Bactec; Becton, Dickinson and Company Sparks, MD) were collected and the patient was started on intravenous (IV) piperacillin-tazobactam.

**FIG 1:**
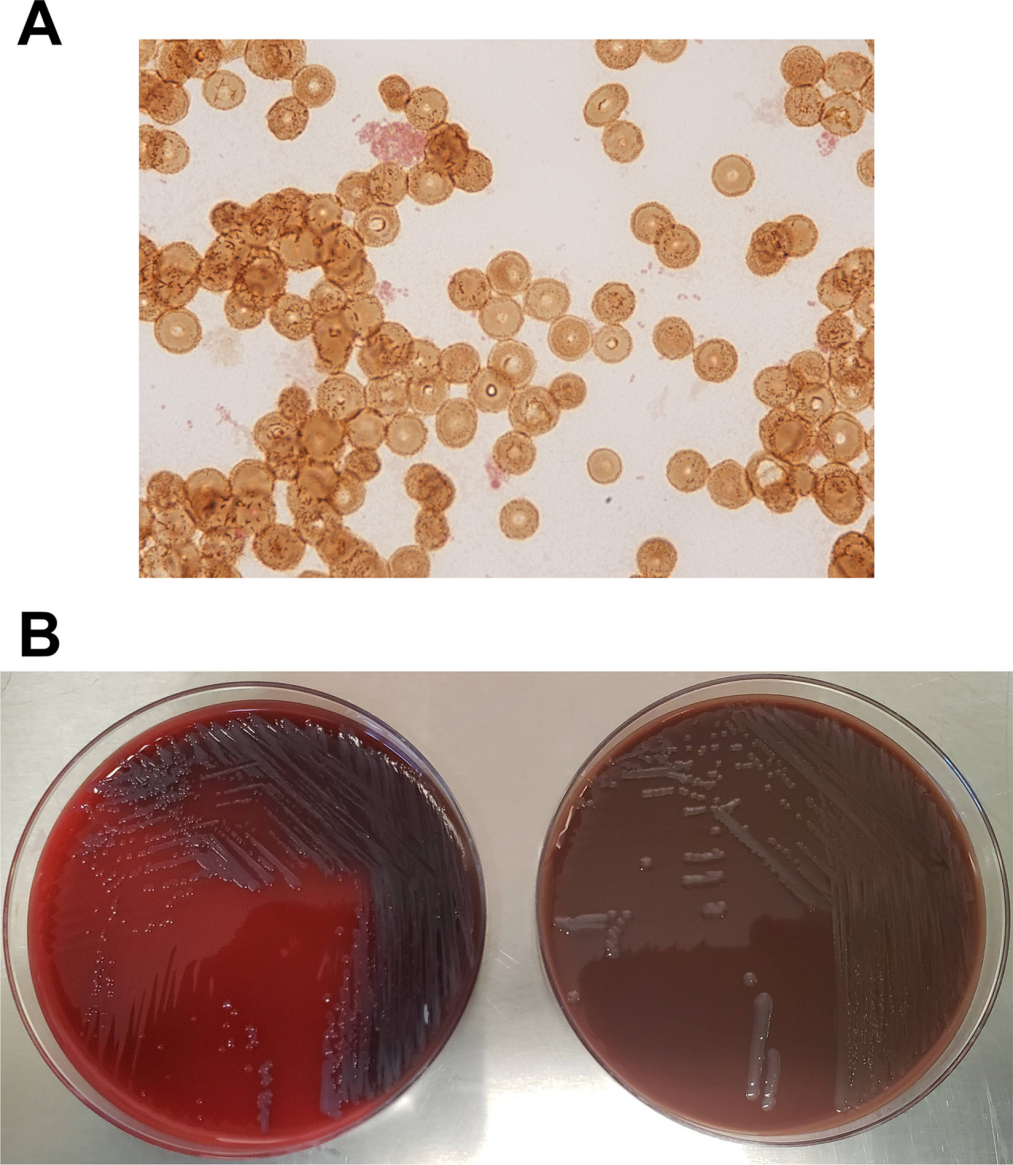
(A) Gram-negative coccobacilli identified from an aerobic blood culture bottle after 3 days of growth. (B) Small gray colonies identified on sheep blood agar (left) and chocolate agar (right) following 2 days of incubation under 5% CO_2_ at 35°C.

After 2 days of incubation, one aerobic bottle grew tiny Gram-negative coccobacilli that could not be identified by the VERIGENE Gram-Negative Blood Culture Test (BC-GN; Luminex, Austin, TX) (Fig. 1A). Broth subcultures plated to sheep blood agar (BAP), chocolate agar (CHOC), MacConkey agar (MAC), and Columbia colistin nalidixic acid agar (CNA) were incubated at 35°C in 5% CO_2_. After 48 h of incubation, small gray colonies were observed on BAP and CHOC (Fig. 1B) and could not be identified by matrix-assisted laser desorption ionization-time of flight (MALDI-TOF) mass spectrometry (Bruker MALDI Biotyper; Bruker, Billerica, MA) or by Vitek 2 GN ID card (bioMérieux, Durham, NC).

Two additional blood culture sets were collected during admission. Three days after collection, both aerobic bottles again grew tiny Gram-negative coccobacilli that tested negative by the BC-GN and subcultures unidentifiable by standard means. Rule-out testing for biothreat agents gave the following reactions: oxidase negative, catalase weakly-positive, urease negative, and β-lactamase positive.

Subsequently, the possibility of a biological threat agent was recognized, resulting in referral of the isolates to Indiana’s Laboratory Response Network (LRN) reference laboratory (Indiana Department of Health Laboratories) for confirmatory testing. There, PCR identified the isolates as Francisella tularensis, and they were forwarded to the Centers for Disease Control and Prevention (CDC) for definitive identification. Serological testing was negative for F. tularensis antibodies, while serum antigen confirmed active infection with a *Blastomyces* spp. (1.36 ng/mL, reportable range: 1.3 to 20 ng/mL). Institutional infection prevention and control services assisted with management of laboratory personnel exposures; implicated laboratorians were prescribed doxycycline and monitored for 14 days. The patient was switched to gentamicin and itraconazole for suspected tularemia and pulmonary blastomycosis. Additional testing by the reference laboratory identified the isolate as F. tularensis subsp. *novicida*, while test results from the CDC ruled out F. tularensis subsp. *tularensis.* Exposure to F. tularensis subsp. *novicida* is believed to have occurred as a result of the patient’s outdoor lifestyle in wooded areas. The patient was successfully treated with a regimen of IV gentamicin followed by 14 days of oral doxycycline.

## DISCUSSION

F. tularensis, a facultative intracellular Gram-negative coccobacillus and the causative agent of tularemia, comprises four subspecies: F. tularensis subspecies *tularensis* (type A, which can be further subtyped into A.I and A.II), F. tularensis subsp. *holarctica* (type B), F. tularensis subsp. *mediasiatica*, and F. tularensis subp. *novicida* (1). Three of the four subspecies are considered Tier 1 select agents by the CDC and United States Department of Agriculture (USDA) Federal Select Agent Program, with F. tularensis subp. *novicida* being the exception (1). Tier 1 agents have the potential to pose severe threats to both human and animal health. Many reservoirs of F. tularensis have been identified, including soil, food, water, and infected animals. Transmission vectors include both arthropods (such as ticks, mosquitos, and deer flies) and vertebrates (classically rabbits and hares), lending the common names of “rabbit fever” or “wild hare disease” (1). F. tularensis is most frequently identified in North America, although it has also been isolated in Europe, Australia, Japan, and China (1, 2). The most commonly isolated subspecies in North America is F. tularensis subsp. *tularensis*, which accounts for roughly 70% of all tularemia cases (1). This is likely due to its low infectious dose, which is <10 CFU for humans (3). In contrast, the infectious doses of F. tularensis subsp. *holartica* (<10^3^ CFU) and F. tularensis subsp. *novicida* (>10^3^ CFU) are higher, and these subspecies tend to cause milder disease in humans and are rarely isolated from patients (4). The infectious dose of F. tularensis subsp. *mediasiatica* in humans has not been reported.

Inhalation is the most common route of infection with F. tularensis and results in respiratory disease, which may be life-threatening. However, other clinical syndromes result when the bacterium penetrates other barriers, including the skin (ulceroglandular), the conjunctivae (oculoglandular), and the upper gastrointestinal tract (oropharyngeal) (1). Ulceroglandular disease is a frequent presentation of tularemia and is marked by ulceration at the inoculation site with satellite inflammatory lymphadenopathy (5). Oculoglandular and oropharyngeal forms present after conjunctival and pharyngeal inoculation, respectively (5). Typhoidal tularemia describes an acute febrile illness that cannot be readily classified as one of the aforementioned syndromes. It typically lacks early signs and symptoms, prominent lymphadenopathy, and has no evidence of a specific inoculation site (5, 6). All forms of tularemia can entail hematogenous spread and acute renal failure (1).

**FIG 2:**
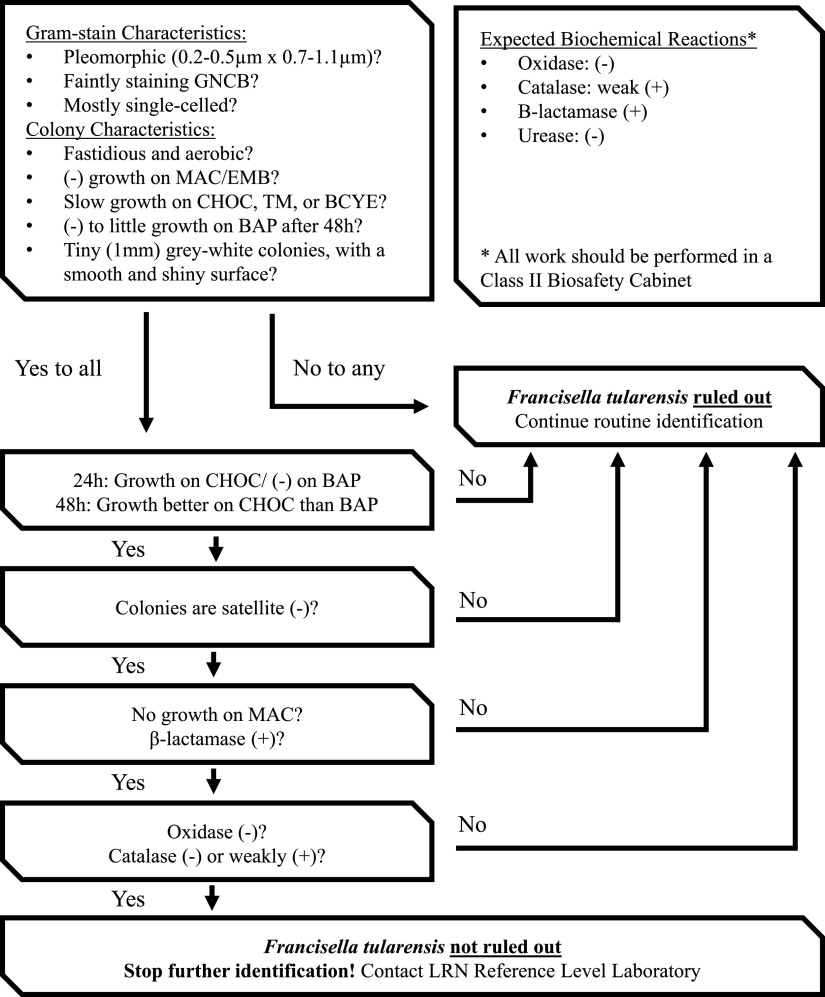
Flow chart algorithm for ruling out Francisella tularensis. (+), positive; (−), negative; BAP, blood agar plate; BCYE, buffered charcoal yeast extract agar; CHOC, chocolate agar; EMB, eosin-methylene blue agar; GNCB, Gram-negative coccobacilli; LRN, Laboratory Response Network; MAC, MacConkey agar; TM, Thayer-Martin agar.

Due to its high infectivity rate, the workup of suspected F. tularensis isolates should be performed in a biosafety cabinet using biosafety level (BSL)-3 precautions and containment along with a strict rule-out algorithm (Fig. 2). F. tularensis is a tiny, pleomorphic, weakly staining Gram-negative coccobacillus (1). It grows poorly on BAP (scant growth of tiny colonies after 48 h), with growth typically better on CHOC (1 to 2 mm gray to grayish-white after >48 h), and does not grow on MAC (1). It is oxidase and urease negative, weakly catalase positive or negative, β-lactamase positive, and nonmotile (1). It is easily mistaken for H. influenzae, although the colonies of F. tularensis fail to exhibit enhanced growth (satellite growth) around S. aureus, indicating there is no requirement for X (hemin) and V (NAD) factors (1). *Francisella* spp. are fastidious in nature, making culture recovery difficult and, in parallel with nonspecific symptoms and an initial mild presentation, diagnosis is often delayed (5, 7). Diagnosis primarily relies on serological and molecular methods (5, 7). Serology is typically not performed in the setting of acute tularemia, as antibodies are often not detectable until 2 to 3 weeks after symptom onset, which may explain our patient’s negative serology results (2). Additionally, IgM and IgG antibodies may remain detectable for several years following resolution of disease and have been reported to cross-react in patients previously infected with Brucella, *Yersinia*, or Salmonella (2). Some molecular methods, such as real-time PCR, are rapid and permit testing of a variety of specimen types (e.g., tissue, sputum, and exudate samples), reducing the time from diagnosis to treatment for the patient (5). However, real-time PCR sensitivity is typically low in blood due to the presence of inhibitors, and it may not identify targets to the subspecies level, which typically requires sequencing (5). Mass spectrometry and other commercial automated methods have been used in the identification of *Francisella* spp. but may be considered as unreliable due to limited spectrum availability with reference databases (7). Additionally, sample preparation for these methods pose an increased risk of exposure and infection to laboratory personnel (7).

Treatment of tularemia includes the use of aminoglycosides (e.g., streptomycin or gentamicin), fluoroquinolones (e.g., ciprofloxacin), or tetracyclines (e.g., doxycycline), with cure rates varying between 60% and 100% depending on the antibiotic used (8). Antimicrobial susceptibility testing (AST) of tularemia-causing strains are typically not performed on a routine basis due to the risk of handling the isolate and a lack of evidence of *in vitro* AST data correlating with treatment failure in tularemia patients (8).

In summary, we present a case on F. tularensis non-subsp. *tularensis* in an immunocompromised patient with a history of blastomycosis. This case illustrates the importance of reviewing standard laboratory practice, as well as educating staff on rule-out algorithms for F. tularensis and other select agents. Initial challenges with cellular morphology interpretation, combined with the reliance on benchtop automated identification systems such as MALDI-TOF, created multiple potentially hazardous incidents for laboratory personnel during culture work-up. Laboratories should include routine competency assessment of personnel to ensure tests results are correctly interpreted, as well as adhering to strict quality control practices pertinent to the relevant tests and their reagents. Laboratory staff who were exposed and followed-up did not go on to develop *Francisella* infection. Fortunately, the F. tularensis isolate described here was not a select agent. Due to the highly infectious nature of F. tularensis, handling of suspected tularemia specimens should always be worked up in a class II biosafety cabinet, ideally in BSL-3 settings.

## SELF-ASSESSMENT QUESTIONS

1. Consequent to a culture of vigilance concerning the handling of suggestive isolates, clinical laboratories initiate BSL-3 precautions and containment, and adherence to strict rule-out algorithms as soon as suspicion of a biothreat agent arises. Nonetheless, according to the CDC and the USDA Federal Select Agent Program, which of the follow identifications confirms that a laboratory has not handled a Tier 1 agent?
  - Francisella tularensis subspecies tularensis
  - Francisella tularensis subspecies holarctica
  - Francisella tularensis subspecies mediasiatica
  - Francisella tularensis subspecies novicida

Answer: d. Francisella tularensis subspecies *novicida* is not regarded as a Tier 1 select agent by the CDC and the USDA Federal Select Agent Program.

2. Given their appearance on Gram stain and their enhanced growth on chocolate agar relative to blood agar, Francisella tularensis subspecies may be mistaken for Haemophilus influenzae. Which of the following is an APHL/LRN/ASM-recommended means of distinguishing these bacteria?
  - A 2,3,5-Triphenyltetrazolium chloride motility test result demonstrating motility
  - A 2,3,5-Triphenyltetrazolium chloride motility test result demonstrating lack of motility
  - An X/V Factor Satellite Test demonstrating growth not isolated to area immediately along Staphylococcus aureus streak or not along sides of X and V factor supplementation
  - An X/V Factor Satellite Test demonstrating growth isolated to area immediately along Staphylococcus aureus streak or along sides of X and V factor supplementation

Answer: c. An X/V Factor Satellite Test demonstrating growth not isolated to the area immediately along the Staphylococcus aureus streak or not along sides of X and V factor supplementation disks/strips differentiates Francisella tularensis from Haemophilus influenzae. While Francisella tularensis is fastidious, it does not require factors X and V for growth; whereas, Haemophilus influenzae does. Francisella tularensis and haemophilus influenzae are both non-motile; therefore, a 2,3,5-Triphenyltetrazolium test result would not be helpful in differentiating between the two bacteria.

3. Seemingly straightforward benchtop tests—such as those for oxidase, catalase, and β-lactamase production—occupy critical decision-points in the APHL/LRN/ASM Francisella tularensis rule-out algorithm. Which of the following measures can be employed to optimize the likelihood that this algorithm correctly routes isolates to LRN Reference Laboratories or back into routine Sentinel Laboratory workflow?
  - Routine competency testing of laboratory personnel to ensure that tests are interpreted correctly
  - Adherence to quality control practices pertinent to these tests and their reagents as outlined by their manufacturers and in accord with internal laboratory practices
  - Notification of the clinical laboratory by care-providers at the earliest indication that a biothreat agent is in the differential
  - All the above

Answer: d. All the above-mentioned measures can be employed to optimize the likelihood that the rule-out algorithm correctly routes suspicious isolates. Vigilance on the part of clinicians and laboratorians alike is necessary to avoid potentially harmful exposures to agents like Francisella tularensis.
